# Impulsivity, anxiety, and alcohol misuse in bipolar disorder comorbid with eating disorders

**DOI:** 10.1186/2194-7511-1-13

**Published:** 2013-08-06

**Authors:** Andrew Jen, Erika FH Saunders, Rollyn M Ornstein, Masoud Kamali, Melvin G McInnis

**Affiliations:** Department of Psychiatry, Penn State Milton S. Hershey Medical Center, Penn State College of Medicine, 500 University Drive, P.O. Box 850, Mail Code HO73, Hershey, PA 17033-0850 USA; University of Michigan Department of Psychiatry, Ann Arbor, MI 48109-5740 USA; University of Michigan Depression Center, Ann Arbor, MI 48105, USA

**Keywords:** Bipolar disorder, Eating disorders, Anxiety disorders, Alcohol use disorders, Impulsivity

## Abstract

**Background:**

Eating disorders (ED) are noted to occur with bipolar disorder (BD), but relationships between additional comorbidities, clinical correlates, and personality factors common to both remain largely unknown.

**Methods:**

Using data from the Prechter Longitudinal Study of Bipolar Disorder, we measured the prevalence and demographic factors of comorbid ED with BD, presence of additional comorbidity of anxiety and substance use disorders, psychosis, suicide attempts, mixed symptoms, childhood abuse, impact of NEO-Personality Inventory (NEO-PI) personality factors, and mood outcome in 354 patients with BD. We analyzed the prevalence of ED using both broad and narrow criteria.

**Results and discussion:**

ED was more common in the Prechter BD sample than the general population, with the majority of those with ED being female. Anxiety disorders, alcohol abuse/dependence, and NEO-PI N5 impulsiveness were independently associated with ED in a multivariable linear regression analysis. BD age at onset was earlier in the ED group than that in the non-ED group and was earlier than the average onset of ED. Anxiety occurred before ED and alcohol use disorders after both BD and ED. Childhood trauma was associated with ED. Impulsivity and anxiety associated with BD may fuel ED and put patients at risk for other impulsivity-related disorders such as alcohol use disorders. ED was associated with more severe and variable moods and more frequent depression. Patients with BD should be regularly screened for ED, anxiety disorders, and alcohol use disorders, and comorbidity should be promptly addressed.

## Background

Bipolar disorder (BD) is an episodic, often disabling disorder of mood, energy, and cognition that has a high level of comorbidity with other psychiatric conditions including anxiety disorders (Simon et al. 2004; Saunders et al. 2012; Potash et al. 2007) and substance abuse (Merikangas et al. 2008; Saunders et al. 2009; Potash et al. 2007) and often results in suicidal ideation and attempts (Chen and Dilsaver 1996; Goodwin and Jamison 2007; Potash et al. 2007) and psychosis (Potash et al. 2007; Potash et al. 2003). Comorbidity with eating disorders (ED), including anorexia nervosa (AN), bulimia nervosa (BN), and binge eating disorder (BED), is a relatively understudied area that may illuminate opportunities for improved treatment for those with both disorders. EDs collectively include an alteration in hunger and satiation cues and overvaluing the role of body size and shape in self-image. Data demonstrate high comorbidity between BD and various EDs, especially among females (Fornaro et al. 2010; McElroy et al. 2011; Brietzke et al. 2011; Seixas et al. 2012). The estimated prevalence of ED in BD has varied widely but ranges between 6% and 27% in BD, many times greater than the population prevalence of 3.6% to 10% (McElroy et al. 2005,2006). EDs are associated with the depressive phases of BD (Mantere et al. 2010), and comorbidity confers greater disease burden (Brietzke et al. 2011). EDs were found to be significantly more common in patients experiencing subsyndromal affective symptoms of BD than patients who were euthymic or fully syndromal (McElroy et al. 2005).

Personality features and temperament may affect the predilection to psychiatric illness and influence the presentation of psychiatric illnesses with regards to comorbidities, biology, and course of illness (Evans et al. 2012a, b; McCrae and Costa 2003; Jylha et al. 2011). When rated with the NEO-Personality Inventory (NEO-PI), BD patients are typically found to have higher scores on neuroticism and openness to experience but lower scores on agreeableness, conscientiousness, and extraversion; and those who were prone to depression were high in neuroticism and low in extraversion, while those prone to mania were higher in extraversion than those prone to depression (Barnett et al. 2011). There may also be differences between bipolar disorder type I and bipolar disorder type II (BP II); in one study, BP II patients scored higher on neuroticism and facets of anxiety, depression, self-consciousness, and vulnerability and lower on extraversion and its facets of positive emotion, as well as conscientiousness and its facets of competence and achievement-striving (Kim et al. 2012).

Both patients with ED and those with BD are vulnerable to negative mood, scoring higher on neuroticism and lower on extraversion and openness to experience on the NEO-PI-R when compared to a representative general population (De Bolle et al. 2011). Patients with EDs and self-injurious behavior appear to be more anxious, more willing to please, and less cheerful, efficient, and ambitious (Claes et al. 2004; Davis and Karvinen 2002). Personality dimensions in patients with EDs may be independent of comorbidity with mood; BN patients had lower well-being compared to people without ED independent of depression, and elevated stress reactivity suggests that BN patients are generally more nervous, upset, and troubled by guilt than other groups of ED patients (Peterson et al. 2010). In the same study, BED patients, like BN patients, scored lower on well-being compared to normal weight comparisons; however, they also score higher on harm avoidance and lower than a normal weight comparison group on positive emotionality (Peterson et al. 2010). Obesity itself has strong associations with emotional distress and depression (Revah-Levy et al. 2011; Petry et al. 2008; de Wit et al. 2010). For example, obese patients have higher depression scores compared to normal weight controls; however, obese people with BED have even higher levels of depression than obese subjects without BED, and they also have a greater tendency towards outward expression of anger (Fau et al. 2003). Patients with BD have elevated rates of being overweight and obese compared to a control population (McElroy et al. 2005). In a systematic review of BD patients, 58% were overweight and 21% had comorbid obesity (Krishnan 2005). Obesity is also associated with an elevated risk of ED psychopathology among BD patients (Wildes et al. 2008). Given the common comorbidity and some shared symptoms such as impulsivity in BD and ED, personality dimensions may overlap in the two disorders. However, while both groups of disorders have been studied separately in the context of personality traits, literature comparing similarities and differences between these two classes is sparse.

To further assess the prevalence, clinical correlates, comorbidities, and personality factors involved in EDs in BD, we systematically examined co-occurring lifetime EDs, other psychiatric comorbidities, and personality traits in patients enrolled in the Prechter Longitudinal Study of Bipolar Disorder. The Prechter Longitudinal Study is a naturalistic, observational study designed to link detailed clinical assessment of psychiatric and medical history, as well as sleep, stress, environmental factors, substance use, personality, and cognition, to clinical outcomes and genetic and biological data for patients being treated with usual clinical care. We hypothesized that EDs would be more common among BD patients in our sample compared to the rates of reported ED in the general population from epidemiological studies, particularly among females, and that the presence of an ED comorbidity would be associated with more severe clinical correlates and additional significant psychiatric comorbidities, particularly anxiety disorders, substance abuse/dependence, and impulsive personality traits.

## Methods

The Prechter Longitudinal Study of Bipolar Disorder is an ongoing naturalistic, observational study of bipolar disorder, conducted with IRB approval (IRBMED HUM000606) at the University of Michigan. De-identified data from the P5 cohort (the participants enrolled between 2005 and 2010) were used in the analysis performed at the Pennsylvania State University College of Medicine, and data were extracted in February 2012. Subjects may enter the study regardless of mood state, and clinical status at baseline is reported in Tables 1 and 2. In this analysis, we used the data from the baseline visit to investigate the lifetime prevalence of ED, comorbidities, and phenotype of BD with ED. Initial evaluation included the Diagnostic Interview for Genetic Studies (DIGS) (Nurnberger et al. 1994), NEO-Personality Inventory, Revised NEO-PI-R (Costa and McCrae 1992), clinician-driven rating scales of mood, questionnaires related to mood and environmental and family stress, personality, drug and alcohol use, neuropsychological testing, physical exam for height and weight, and a blood draw for genetics and saliva collection for cortisol. A best estimate process by at least two independent psychiatrists or psychologists was used to determine diagnoses (Leckman et al. 1982). For this analysis, the frequency, clinical correlates, and personality dimensions of ED was determined in BD individuals (bipolar disorder type I, *n* = 263; bipolar disorder type II, *n* = 59; bipolar disorder NOS, *n* = 23; schizoaffective disorder, bipolar type, *n* = 9). Diagnosis and features of illness were obtained from the DIGS and the best estimate process; personality factors including neuroticism, extroversion, openness, agreeableness, conscientiousness, and the N5 facet score for impulsiveness were obtained from the NEO-PI-R. In addition, some subjects completed the Barrett Impulsiveness Scale (BIS) (Barratt 1975) (*n* = 174) and the childhood trauma questionnaire (CTQ) (Bernstein et al. 1997,1994) (*n* = 272). Self-rated questionnaires, including the Patient Health Questionnaire (PHQ-9) (Kroenke et al. 2001) and the Altman Self-Rating Mania Scale (ASRM) (Altman et al. 1997), were completed every 2 months for 2 years (*n* = 277). Baseline depressive symptoms were measured using the Hamilton Depression Rating Scale (Hamilton 1960), and manic symptoms were measured with the Young Mania Rating Scale (Young et al. 1978). Mood outcomes were characterized by severity, variability, and frequency of clinically significant symptoms of depression or mania through the duration of follow-up, which differed for each participant. The severity of depression outcome was defined for each individual by the maximum PHQ-9 score over follow-up; the severity of mania outcome was defined for each individual by the maximum ASRM score over follow-up. The variability of the depression outcome was defined for each individual by the standard deviation in PHQ-9 scores over the duration of follow-up; the variability of the mania outcome was defined by the standard deviation in ASRM scores over the duration of follow-up. The frequency of clinically significant depressive or manic symptoms was defined as the proportion of the follow-up period that the individual had a PHQ-9 or ASRM score over 5.

**Table 1:** **Demographics/description of sample**

	Non-ED (***n*** = 291)	ED (***n*** = 63)	***p*** value
	Mean (SD)	Mean (SD)	
Age at interview	41.02 (13.20)	36.60 (11.80)	.02
BD age at onset	18.41 (7.90)	15.08 (6.70)	2 × 10^−3^
BMI	28.95 (6.30)	29.78 (8.20)	.37
Personality			
Neuroticism	61.97 (14.07)	67.40 (12.77)	0.1
Extraversion	49.62 (11.81)	50.76 (11.88)	.49
Openness	56.84 (11.71)	61.06 (11.78)	.04
Agreeableness	49.15 (12.59)	43.83 (15.33)	4 × 10^−3^
Conscientiousness	42.81 (13.20)	39.95 (14.13)	.13
N5 impulsiveness	57.12 (10.71)	63.97 (11.06)	<1 × 10^−3^
BIS total (*n* = 143/31)	66.00 (13.05)	70.81 (14.00)	.07
CTQ Total (*n* = 222/50)	48.19 (17.60)	57.18 (20.54)	2 × 10^−3^
Outcome measures (*n* = 228/49)			
Depression severity	14.2 (7.2)	18.6 (5.9)	<1 × 10^−3^
Depression frequency	.59 (.37)	.78 (.26)	<1 × 10^−3^
Depression variability	3.9 (2.4)	4.9 (2.4)	.01
Mania severity	7.7 (4.3)	8.5 (4.6)	.22
Mania frequency	.25 (.28)	.23 (.24)	.53
Mania variability	2.4 (1.4)	2.9 (1.8)	.03
Mixed symptoms frequency	.43 (.50)	.51 (.51)	.31

**Table 2:** **Clinical description of samples**

	***n*** (% of Non-ED)	***n*** (% of ED)	***p*** value
	Mean (SD)	Mean (SD)	
Female	185 (64)	52 (83)	4 × 10^−3^
Bipolar disorder type I	218 (75)	45 (71)	
Bipolar disorder type II	48 (17)	11 (18)	
Bipolar disorder NOS	18 (6)	5 (8)	
Schizoaffective disorder, bipolar type	7 (2)	2 (3)	.92
Mood state at baseline			
Euthymic	135 (53)	13 (23)	
Depressed	88 (34)	34 (60)	
Manic/hypomanic/mixed	34 (13)	10 (18)	<1 × 10^−3^
All narrow ED (% of all)	-	38 (11)	
Narrow anorexia (% of all)	-	6 (2)	
Narrow bulimia (% of all)	-	13 (4)	
Binge eating disorder (% of all)	-	29 (8)	
Broad anorexia (% of all)	-	16 (5)	
Broad bulimia (% of all)	-	36 (10)	
Any anxiety disorder	102 (35)	40 (64)	<1 × 10^−3^
Obsessive compulsive disorder	10 (3)	10 (16)	<1 × 10^−3^
Panic disorder	64 (22)	26 (41)	.02
Social phobia	33 (11)	17 (34)	1 × 10^−3^
Specific phobia	24 (8)	11 (18)	.03
Agoraphobia	11 (4)	3 (5)	.72
Alcohol use disorder	120 (41)	38 (60)	6 × 10^−3^
Drug use disorder	98 (34)	27 (43)	.17
Psychosis	157 (58)	30 (49)	.22
Suicide attempts	113 (40)	25 (40)	.91
Mixed symptoms	109 (42)	34 (60)	.02

### Diagnoses and assessments

Subjects were classified into ED diagnostic categories using narrow and broad phenotypes. The narrow phenotypes included AN, BN, and BED as diagnosed using Diagnostic and Statistical Manual of Mental Disorders IV, Text Revision (DSM-IV-TR) criteria. Broader diagnoses for AN and BN were created consistent with proposed revisions to ED diagnosis for DSM-5. A broad AN diagnosis was determined if the patient at one point weighed less than somebody thought they ought to weigh, lost weight on purpose, and had a lowest body mass index (BMI) below 17.5. A broad BN diagnosis was made if the patient had binged, felt out of control while binging, and engaged in compensatory behaviors (including typical purging behaviors, as well as fasting and overexercising behaviors). Time criteria were excluded from broad BN diagnoses because the DIGS assessed the frequency of behaviors only based on DSM-IV criteria. Alcohol use disorders encompassed lifetime history of alcohol abuse and alcohol dependence, as diagnosed by the DIGS, and validated through the best estimate process. Anxiety disorders included lifetime history of social phobia, specific phobia, obsessive compulsive disorder (OCD), panic disorder, and agoraphobia, and were also diagnosed by the DIGS and validated through the best estimate process. Age at onset of the whole anxiety disorders group was calculated using the youngest age at onset for those with multiple anxiety disorders.

### Statistical analysis

Age at interview, age at onset, and BMI were compared using *t* tests. Chi-square was used to compare categorical variables (comorbid panic disorder, OCD, social phobia, specific phobia, alcohol use disorders and drug use disorders, history of psychosis, suicide attempts, and mixed symptoms). Personality scores from the NEO-PI-R were compared using *t* tests, as were scores from the BIS and CTQ. Factors that differed between ED and non-ED groups were tested for correlation to ED using chi-square between two categorical variables and bivariate correlations for continuous variables using Pearson's correlation coefficient. Those factors that were individually associated with ED were included in multivariable logistic regression models based on domain, one in the domain of comorbidities and features of BD illness and a second model for personality factors. A backward selection process was used to identify significant predictors. A unified multivariable logistic regression model was generated with backward selection to determine the best predictors of ED from both sets of variables. All models were corrected for sex and age. Outcome variables including depression severity, frequency, and variability; manic severity, frequency, variability; and mixed symptom frequency were compared between groups using *t* tests. Multivariable linear regression models accounting for age, sex, and baseline mood status were created to determine the predictors of mood outcome.

## Results

### Eating disorder rates in the Prechter BD sample

As shown in Tables 1 and 2, the analysis of the dataset found EDs (*n* = 63) to be more prevalent in the Prechter BD (PBD) sample (*n* = 354) than in the general population. All narrow ED diagnoses were higher than the population rates: narrow AN had a prevalence of 2% in the PBD sample compared to a population norm of 0.3%, narrow BN was 13% compared to 1.0%, and BED was 8% compared to 2.6. Broad AN and broad BN had prevalence rates of 5% and 10% in the PBD sample, respectively, but general population prevalence is currently unknown. Total ED prevalence was 18%, which includes BD patients who meet narrow and/or broad diagnostic criteria for ED. Eighty-three percent of the ED group was female.

### Clinical description of the ED and non-ED Prechter BD sample

At study baseline, individuals with ED were more likely to be symptomatic and less likely to be euthymic than those without ED (depression, 60% vs 34%; mania/hypomania/mixed, 18% vs 13%; euthymic, 23% vs 53%; *p* = 1 × 10^−3^) (Table 2). Individuals with ED were younger at the age of interview (36 ± 12 years vs 41 ± 13 years) and had a lower age at onset of BD by a mean of 3 years than those without ED (15 ± 7 years vs 18 ± 8 years) (Table 1). There was no difference between those with and without ED in time from first mood symptoms to first being seen for psychiatric care (4.1 ± 10.1 years vs 3.6 ± 9.4 years, *p* = .74). Those with ED and those without ED had no significant difference in current BMI (Table 1). Several personality factors differed significantly between the ED and non-ED groups in the PBD sample (Table 1). Neuroticism, openness to experience, and N5 impulsiveness were higher and agreeableness was lower in the ED group than in the non-ED group, and the ED group also had more childhood trauma as measured by the CTQ (Table 1).

As reported in Table 1, during the follow-up period of the longitudinal study, individuals with ED were more likely to have more severe depression (*p* < 1 × 10^−3^), as measured by maximum scores on the PHQ-9 self-rating depression scale than those without ED. Individuals with ED more frequently had clinically significant depressive symptoms measured by a score of >5 on the PHQ-9 (*p* < 1 × 10^−3^) and had more variability in depressive symptoms than those without ED (*p* = .01). Individuals with ED also had more variability in manic symptoms than those without ED (*p* = .03), but the ED group did not have more severe or frequent mania or mixed symptoms. The comparisons reported above remained significant after accounting for age, sex, and baseline mood symptoms, except for depression variability which was then significant at a trend level (*p*=.09).

BD subjects with ED had higher rates of comorbid anxiety disorders, alcohol abuse/dependence, and mixed symptoms than BD subjects without ED (Table 2). The overall rate of comorbid anxiety disorders was 64% for the ED group and 35% for the non-ED group. Individual anxiety disorders present a higher rate in the ED group compared to the non-ED group for OCD (16% vs 3%), social phobia (34% vs 11%), specific phobia (18% vs 8%), and panic disorder (41% vs 22%), but not agoraphobia (5% vs 4%). BD patients with ED also had a higher rate of diagnosis with alcohol abuse or dependence (60% vs 41%). Of the clinical correlates analyzed (history of psychosis, suicide attempts, or mixed symptoms), only mixed symptoms was statistically different between ED and non-ED groups (60% vs 42%).

**Figure 1:**
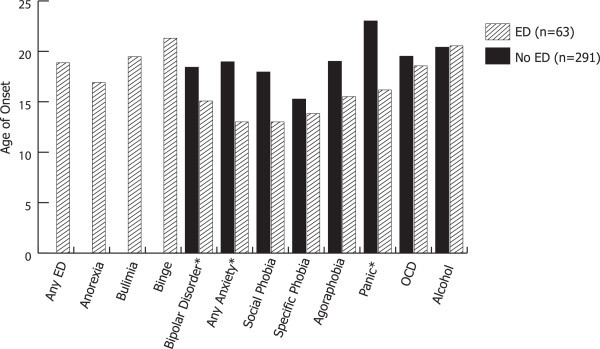
**Age at onset of BD, EDs, anxiety disorders, and alcohol use disorder.** Asterisk indicates that differences between those with and without ED are statistically significant.

The onset of ED occurred on average at 19 ± 9 years of age for the entire ED group, but the onset of AN was the youngest at 17 ± 6 years, followed by BN at 20 ± 7 years and BED at 21 ± 11 years. Age at onset of anxiety disorders was significantly lower in the ED group than in the non-ED group, but the age at onset of alcohol use disorders did not differ (Figure 1, Table 3). Anxiety in the ED group most commonly preceded the onset of ED, but alcohol abuse/dependence followed the onset of ED (Figure 1, Table 3). Of the specific anxiety disorders, only panic disorder had a significantly lower age at onset in the ED group (onset at 16 ± 11 years compared to 23 ± 10 years in the non-ED group). However, social phobia had an average onset at 13 ± 7 years compared to 18 ± 11 years and specific phobia at 14 ± 9 years compared to 15 ± 13 years. These differences may be significant with a larger sample size. The onset of OCD was similar in both groups (19 ± 14 years vs 20 ± 10 years).

**Table 3:** **Age at onset of BD, anxiety disorders, and alcohol use disorder in those with and without ED**

	Non-ED (***n*** = 291)	ED (***n*** = 63)	ED before comorbidity
BD*	18.41 (7.90)	15.08 (6.70)	
ED	-	18.86 (9.30)	-
Anorexia	-	16.90 (5.91)	
Bulimia	-	19.46 (6.57)	
Binge	-	21.30 (10.93)	
Anxiety*	18.95 (11.28)	13.00 (7.75)	18%
Social phobia	17.94 (11.11)	13.00 (7.38)	
Specific phobia	15.25 (12.56)	13.83 (9.34)	
Agoraphobia	19.00 (12.15)	15.50 (3.54)	
Panic*	23.00 (10.09)	16.17 (10.83)	
OCD	19.50 (9.77)	18.55 (14.40)	
Alcohol	20.39 (6.45)	20.54 (7.36)	65%

^*^ *p* < .05.

### Factors associated with ED

In bivariate analyses, having a history of anxiety disorders, alcohol use disorders, or mixed symptoms were associated with ED (Table 4). Neuroticism, openness, agreeableness, and NEO-PI N5 impulsiveness were also associated at the bivariate level with EDs (Table 4). In a subset of the sample, BIS impulsivity was associated with ED at the trend level, and in a separate subset, CTQ trauma was also associated with EDs (Table 4).

**Table 4:** **Associations of illness, comorbidity, and personality factors with eating disorders in the bipolar sample**

Domain	Bivariate	Analysis by domain^a^	Unified^b^
	***r*** (***p*** value)	OR (CI)	OR (CI)
Comorbidities and features of illness			
Anxiety	*p* < 1 × 10^−3^	2.5 (1.35, 4.76)	2.37 (1.30, 4.34)
Alcohol	*p* = 6 × 10^−3^	2.20 (1.18, 4.11)	2.07 (1.14, 4.34)
Mixed symptoms	*p* = .02	1.76 (.94, 3.32)	-
Personality factors			
Neuroticism	.15 (.01)	-	-
Openness	.13 (.01)	-	-
Agreeableness	−.15 (4 × 10^−3^)	-	-
Conscientiousness	−.08 (.13)	-	-
N5 impulsiveness	.24 (<1 × 10^−3^)	1.07 (1.04, 1.10)	1.05 (1.02, 1.09)
BIS impulsivity (*n* = 174)^d^	.14 (.07)	-	-
CTQ trauma (*n* = 272)^d^	.19 (2 × 10^−3^)	1.03 (1.01, 1.04)	-

^a^Multivariable linear regression analysis were performed with backward selection within domain, adjusted for age and sex. ^b^Multivariable linear regression analysis were performed with backward selection with all factors that are significant within domains, adjusted for age and sex. ^c^OR when analyzed by standard deviation, 1.502 (1.193, 1.892). ^d^Multivariable linear regression analysis performed for BIS and CTQ separately due to a subset of the sample having these measures.

**Figure 2:**
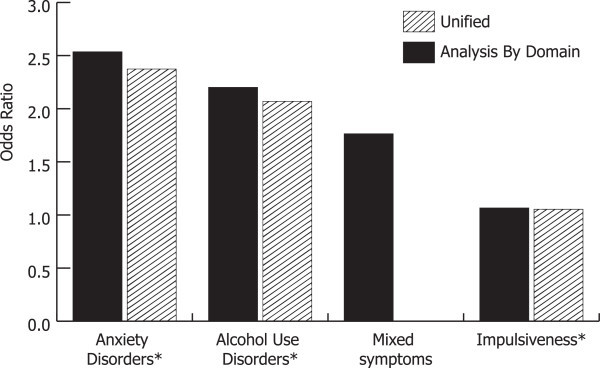
**Factors that are associated with ED in the PBD sample.** By domain and unified analysis, controlled for age and sex. Asterisk indicates that variables were independently associated with an increased risk for ED in the unified analysis.

The above factors were included each in a regression analysis that controlled for age and sex. As shown in Figure 2 and Table 4, anxiety disorders, alcohol use disorders, and mixed symptoms were analyzed together as features of illness domain. Anxiety disorders and alcohol use disorders emerged as independent predictors in this domain, with anxiety having the highest risk (OR 2.5, CI 1.4, 4.8), followed by alcohol use disorders (OR 2.2, CI 1.2, 4.1). In the domain of personality factors, N5 impulsiveness emerged as an independent predictor (OR 1.1, CI 1.0, 1.1). BIS impulsivity was analyzed in a separate regression analysis, controlling for age and sex, and did not emerge as significant. CTQ trauma was significant in an independent analysis controlling for age and sex (OR 1.0, CI 1.0, 1.0).

Anxiety disorder, alcohol use disorders, N5 impulsiveness, and mixed symptoms were included in a multivariable unified model, controlling for age and sex, to encompass both domains. Anxiety disorders (OR 2.4, CI 1.3, 4.4), alcohol use disorders (OR 2.1, CI 1.1, 4.3), and N5 impulsiveness (OR 1.1, CI 1.0, 1.1) each emerged as being independently associated with ED (Figure 2, Table 4).

## Discussion

We found that BD comorbid with ED was strongly associated with other comorbidities, including anxiety disorders, alcohol use disorders, and impulsiveness when accounting for age and sex. Our findings of high lifetime rates of EDs in a sample of BD patients are consistent with the findings of previous studies, suggesting that ED and BD are comorbid at a rate higher than what would be predicted through chance alone (McElroy et al. 2011; McElroy et al. 2006,2005; Brietzke et al. 2011; Seixas et al. 2012; Wildes et al. 2008). As expected, comorbidity with ED was associated with female gender and earlier age at onset of BD by an average of 3.3 years. We found, however, a striking difference between the patterns of onset with anxiety compared to alcohol use comorbidities. The age at onset of anxiety disorders in the ED group was almost 6 years before non-ED population, specifically social phobia and specific phobia. Anxiety disorders occurred before the average onset of ED, even the earliest of the ED diagnoses, AN. BN and BED occurred in late adolescence and coincided with the onset of alcoholism. However, the age at onset of alcohol use disorders was very similar to the non-ED population. We therefore postulate that individuals with a predilection to BD and ED have a high likelihood of anxiety in adolescence, followed by development of BD, then ED, and then alcohol use disorder. Alcohol use disorder is often a coping mechanism for anxiety and mood symptoms and is particularly a high risk for females with BD and anxiety disorders (Saunders et al. 2012). Understanding this sequence of vulnerability can be useful in clinically caring for adolescents, particularly females, with anxiety disorders and bipolar disorder.

The NEO-PI N5 facet of impulsiveness was associated with ED after accounting for age, sex, comorbid anxiety, and alcohol use disorders. Impulsivity in general is a common trait in BD (Lombardo et al. 2012) and is associated with worse functioning and greater stress, as well as nicotine addiction (Lombardo et al. 2012; Molz et al. 2013; Heffner et al. 2012; Henna et al. 2013). Though BD patients with comorbid alcohol use disorders have increased trait impulsivity compared to BD patients without alcohol misuse (Nery et al. 2013), in our analysis, impulsivity was independently associated with ED. Systematic review has found that BED patients have greater reward sensitivity, one of the two components of impulsivity (Schag et al. 2013). High impulsivity is also correlated with poor decision-making behaviors in binge/purge-type EDs (Garrido and Subira 2013), and as with BD, greater impulsivity is predictive of higher rates of alcohol misuse among patients with AN (Baker et al. 2013) or a greater tendency towards self-harming behavior in AN and BN patients (Davis and Karvinen 2002). The overlap in impulsivity between BD and ED suggests a possibly personality trait connection between these two spectrums of disorders.

Childhood trauma is also associated with both BD and ED and was found to be associated in our study. The CTQ is a self-report form commonly used to assess childhood neglect and abuse on a physical, sexual, and emotional level (Bernstein et al. 1997). Total scores on the CTQ are higher for BD than for normal controls, and further analysis reveals that emotional abuse in particular is associated with BD, with a suggestive dose effect (Etain et al. 2010). Childhood trauma is also associated with worse cognitive function (Aas et al. 2012), as well as greater aggression traits in bipolar patients (Garno et al. 2008), suggesting that trauma plays a role in clinical outcomes and disease burden of BD (Daruy-Filho et al. 2011). Similarly, EDs are sometimes associated with childhood trauma or maltreatment; in particular, AN binging/purging-type patients score higher on the CTQ (Jaite et al. 2012), and BED patients report higher rates of emotional abuse and emotional neglect than controls. This was associated with greater levels of depression (Allison et al. 2007).

We found a difference between those with and without ED regarding longitudinal outcome, including more severe and frequent depressive symptoms and more variability in depressive and manic symptoms, which can be conceptualized as more mood instability. We conclude from these data that having comorbid ED may be associated with more mood instability. However, we cannot conclude that the mood instability is caused by ED from these data because we do not have measures of active ED symptoms during the follow-up period, and there may be additional moderating factors not accounted for in the current analysis.

Contrary to other studies, our study found no significant difference in current BMI in BD patients with ED and without ED (Wildes et al. 2007,2008; McElroy et al. 2011). It may reflect the phase of illness; most of our BD patients were in remission from ED, with a BMI that had normalized. Another factor is the treatment for BD; many of the pharmacologic treatments increase BMI through increased appetite, and BD patients are at risk for obesity partly due to being on treatment. In addition, for analysis, we grouped all ED categories together, rather than examining each ED individually, which might mask the differences in BMI between ED and non-ED groups.

Findings should be viewed in light of several methodological limitations. First, this study is an observational study and thus the correlations found do not represent an attempt to discuss the nature of causality for the correlations found herein but simply that these correlations exist. Second, definitions of broad AN and BN do not exactly represent the definitions of AN and BN as described in the DSM-5. Efforts were made to approximate the proposed changes for ED diagnosis, but the current version of the DIGS does not contain questions for all criteria necessary to exactly replicate the proposed DSM-5 criteria. Third, the subset of patients with ED was small, and not all differences that are truly present may have represented in this study. Fourth, subjects with data in the PBD sample may not represent BD patients in the general population, in particular, those that are not under medical treatment. Therefore, the results of this study may not be generalizable to all bipolar spectrum patients. Finally, while a broad range of comorbidities are discussed, formal DSM-based personality disorder diagnoses were not evaluated, as personality dimensions were evaluated using a dimensional approach in the five-factor model and specific evaluation of impulsivity. This study was designed prior to the considerations of the Research Domain Criteria (RDoC) (Cuthbert and Insel 2013); subsequently, the proposed systems and processes of RDoC are not addressed. Considerable efforts were made, however, to capture transdiagnostic dimensional data, including personality structure, and vulnerability factors such as exposure to trauma.

However, this study also has several strengths. The PBD repository includes a wide variety of detailed information from over 300 patients with BD. Structured interviews, clinician-administered questionnaires, and self-administered questionnaires were used to diagnose a wide spectrum of both BD and EDs, including ED categories that closely approximate proposed DSM-5 revisions to AN and BN. The highly detailed information on these patients also permits accurate assessment and systemic evaluation of the relationship between BD and demographic factors, clinical correlates, ED comorbidity, additional psychiatric comorbidities such as anxiety disorders or substance abuse/dependence, personality factors, and childhood trauma.

Our current nosological system divides and categorizes diagnoses in such a way that many patients with bipolar disorder have concurrent diagnoses. These are classified as ‘comorbidities’ and are discussed as such. In this system, we are limited to describing phenomenology, as biological findings associated with psychopathology do not map neatly to the nosology. At least two hypotheses of what is occurring biologically are plausible: each clinical state is the result of a biological process and patients with co-morbidities have several biological processes occurring at once, or most of the time, patients have one biological process that causes psychiatric disturbance, and the clinical phenotypes that are exhibited are a result of that one process, perhaps interacting with the environment (prenatal, shared, and non-shared). One way to bridge the gap is through careful phenotyping of clinical syndromes that can then be used to probe biological and genetic underpinnings of disease.

The findings have several important clinical implications that must be viewed with caution in light of the observational nature of our study. First, high rates of co-occurrence of BD and EDs suggest that screening for EDs should be undertaken when performing comprehensive evaluations of BD, and future assessment should include careful observation for the development of ED, especially among adolescent and young adult female patients. Second, because of the younger age at onset of BDs in ED patients, patients presenting early with BD should be screened repeatedly for signs of ED, and patients presenting with ED should be screened early for signs of BD. Third, in patients with both BD and ED, assessment of comorbid alcohol abuse/dependence and anxiety disorders such as panic disorder, OCD, social phobia, and specific phobia should take place. In patients with both BD and ED, approximately 64% will have a comorbid anxiety disorder and 60% will have comorbid alcohol abuse/dependence, prompting serious consideration of both comorbidities when evaluating BD patients with ED. Anxiety often precedes ED, and alcohol abuse/dependence often follows onset of ED; so, screening should address both the current presence of anxiety symptoms and future development of alcohol abuse/dependence. Impulsiveness, anxiety disorders, and alcohol use disorders aggregate together and indicate higher risk of comorbid psychopathology. Prospective studies are needed to determine whether differential variation in treatment based on psychiatric comorbidities produces better outcomes in BD or ED patients.

## Conclusions

Eating behavior is clearly related to mood disorders and its manifestations and becomes disordered at specific thresholds of weight, actions, and compensatory behaviors. BD with its mercurial and impulsive phenomenology fuels ED behaviors resulting in amplification of the illnesses and compromising the course and outcomes of both. The complexity of the comorbidity is reflected in the diversity of contributing factors that range from personality features and self-image to life story exposure of traumatic experiences. Complications include the side effects of many treatments that include appetite and weight changes. Prospective studies aimed at the BD individual with disordered eating behavior will be useful in determining the nature of the relationship between BD and ED and appropriate interventions in comorbid conditions.

## References

[CR1] Aas M, Steen NE, Agartz I, Aminoff SR, Lorentzen S, Sundet K, Andreassen OA, Melle I (2012). Is cognitive impairment following early life stress in severe mental disorders based on specific or general cognitive functioning?. Psychiatry Res.

[CR2] Allison KC, Grilo CM, Masheb RM, Stunkard AJ (2007). High self-reported rates of neglect and emotional abuse, by persons with binge eating disorder and night eating syndrome. Behav Res Ther.

[CR3] Altman EG, Hedeker D, Peterson JL, Davis JM (1997). The Altman self-rating mania scale. Biol Psychiatry.

[CR4] Baker JH, Thornton LM, Strober M, Brandt H, Crawford S, Fichter MM, Halmi KA, Johnson C, Jones I, Kaplan AS, Klump KL, Mitchell JE, Treasure J, Woodside DB, Berrettini WH, Kaye WH, Bulik CM (2013). Temporal sequence of comorbid alcohol use disorder and anorexia nervosa. Addict Behav.

[CR5] Barnett JH, Huang J, Perlis RH, Young MM, Rosenbaum JF, Nierenberg AA, Sachs G, Nimgaonkar VL, Miklowitz DJ, Smoller JW (2011). Personality and bipolar disorder: dissecting state and trait associations between mood and personality. Psychol Med.

[CR6] Barratt ES (1975). Barratt Impulsiveness Scale.

[CR7] Bernstein DP, Fink L, Handelsman L, Foote J, Lovejoy M, Wenzel K, Sapareto E, Ruggiero J (1994). Initial reliability and validity of a new retrospective measure of child abuse and neglect. Am J Psychiatry.

[CR8] Bernstein DP, Ahluvalia T, Pogge D, Handelsman L (1997). Validity of the Childhood Trauma Questionnaire in an adolescent psychiatric population. J Am Acad Child Adolesc Psychiatry.

[CR9] Brietzke E, Moreira CL, Toniolo RA, Lafer B (2011). Clinical correlates of eating disorder comorbidity in women with bipolar disorder type I. J Affect Disord.

[CR10] Chen YW, Dilsaver SC (1996). Lifetime rates of suicide attempts among subjects with bipolar and unipolar disorders relative to subjects with other Axis I disorders. Biol Psychiatry.

[CR11] Claes L, Vandereycken W, Vertommen H (2004). Personality traits in eating-disordered patients with and without self-injurious behaviors. J Pers Disord.

[CR12] Costa PT, McCrae RR (1992). Revised NEO Personality Inventory (NEO PI-R) and NEO Five-Factor Inventory (NEO-FFI) Professional Manual.

[CR13] Cuthbert BN, Insel TR (2013). Toward the future of psychiatric diagnosis: the seven pillars of RDoC. BMC Med.

[CR14] Daruy-Filho L, Brietzke E, Lafer B, Grassi-Oliveira R (2011). Childhood maltreatment and clinical outcomes of bipolar disorder. Acta Psychiatr Scand.

[CR15] Davis C, Karvinen K (2002). Personality characteristics and intention to self-harm: a study of eating disordered patients. Eat Disord.

[CR16] De Bolle M, De Clercq B, Pham-Scottez A, Mels S, Rolland J-P, Guelfi JD, Braet C, De Fruyt F (2011). Personality pathology comorbidity in adult females with eating disorders. J Health Psychol.

[CR17] de Wit L, Luppino F, van Straten A, Penninx B, Zitman F, Cuijpers P (2010). Depression and obesity: a meta-analysis of community-based studies. Psychiatry Res.

[CR18] Etain B, Mathieu F, Henry C, Raust A, Roy I, Germain A, Leboyer M, Bellivier F (2010). Preferential association between childhood emotional abuse and bipolar disorder. J Trauma Stress.

[CR19] Evans SJ, Kamali M, Prossin AR, Harrington GJ, Ellingrod VL, McInnis MG, Burant CF (2012). Association of plasma omega-3 and omega-6 lipids with burden of disease measures in bipolar subjects. J Psychiatr Res.

[CR20] Evans SJ, Prossin AR, Harrington GJ, Kamali M, Ellingrod VL, Burant CF, McInnis MG (2012). Fats and factors: lipid profiles associate with personality factors and suicidal history in bipolar subjects. PloS One.

[CR21] Fau FS, Abbate-Daga G, Fau A-DG, Piero A, Fau PA, Leombruni P, Fau LP, Rovera GG, Rovera GG (2003). Dropout from brief psychotherapy within a combination treatment in bulimia nervosa: role of personality and anger. Psychother Psychosom.

[CR22] Fornaro M, Perugi G, Gabrielli F, Prestia D, Mattei C, Vinciguerra V, Fornaro P (2010). Lifetime co-morbidity with different subtypes of eating disorders in 148 females with bipolar disorders. J Affect Disord.

[CR23] Garno JL, Gunawardane N, Goldberg JF (2008). Predictors of trait aggression in bipolar disorder. Bipolar Disord.

[CR24] Garrido I, Subira S (2013). Decision-making and impulsivity in eating disorder patients. Psychiatry Res.

[CR25] Goodwin FK, Jamison KR (2007). Manic-Depressive Illness: Bipolar Disorders and Recurrent Depression.

[CR26] Hamilton M (1960). A rating scale for depression. J Neurol Neurosurg Psychiatry.

[CR27] Heffner JL, Fleck DE, DelBello MP, Adler CM, Strakowski SM (2012). Cigarette smoking and impulsivity in bipolar disorder. Bipolar Disord.

[CR28] Henna E, Hatch JP, Nicoletti M, Swann AC, Zunta-Soares G, Soares JC (2013). Is impulsivity a common trait in bipolar and unipolar disorders?. Bipolar Disord.

[CR29] Jaite C, Schneider N, Hilbert A, Pfeiffer E, Lehmkuhl U, Salbach-Andrae H (2012). Etiological role of childhood emotional trauma and neglect in adolescent anorexia nervosa: a cross-sectional questionnaire analysis. Psychopathology.

[CR30] Jylha P, Mantere O, Melartin T, Suominen K, Vuorilehto M, Arvilommi P, Holma I, Holma M, Leppämäki S, Valtonen H, Rytsälä H, Isometsä E (2011). Differences in temperament and character dimensions in patients with bipolar I or II or major depressive disorder and general population subjects. Psychol Med.

[CR31] Kim B, Lim J-H, Kim SY, Joo YH (2012). Comparative study of personality traits in patients with Bipolar I and II disorder from the Five-Factor Model Perspective. Psychiatry Investig.

[CR32] Krishnan KRR (2005). Psychiatric and medical comorbidities of bipolar disorder. Psychosom Med.

[CR33] Kroenke K, Spitzer RL, Williams JB (2001). The PHQ-9: validity of a brief depression severity measure. J Gen Intern Med.

[CR34] Leckman JF, Sholomskas D, Thompson WD, Belanger A, Weissman MM (1982). Best estimate of lifetime psychiatric diagnosis: a methodological study. Arch Gen Psychiatry.

[CR35] Lombardo LE, Bearden CE, Barrett J, Brumbaugh MS, Pittman B, Frangou S, Glahn DC (2012). Trait impulsivity as an endophenotype for bipolar I disorder. Bipolar Disord.

[CR36] Mantere O, Isometsa E, Ketokivi M, Kiviruusu O, Suominen K, Valtonen HM, Arvilommi P, Leppämäki S (2010). A prospective latent analyses study of psychiatric comorbidity of DSM-IV bipolar I and II disorders. Bipolar Disord.

[CR37] McCrae RR, Costa PT (2003). Personality in Adulthood: A Five-Factor Theory Perspective.

[CR38] McElroy SL, Kotwal R, Keck PE, Akiskal HS (2005). Comorbidity of bipolar and eating disorders: distinct or related disorders with shared dysregulations?. J Affect Disord.

[CR39] McElroy SL, Kotwal R, Keck PE (2006). Comorbidity of eating disorders with bipolar disorder and treatment implications. Bipolar Disord.

[CR40] McElroy SL, Frye MA, Hellemann G, Altshuler L, Leverich GS, Suppes T, Keck PE, Nolen WA, Kupka R, Post RM (2011). Prevalence and correlates of eating disorders in 875 patients with bipolar disorder. J Affect Disord.

[CR41] Merikangas KR, Herrell R, Swendsen J, Rossler W, Ajdacic-Gross V, Angst J (2008). Specificity of bipolar spectrum conditions in the comorbidity of mood and substance use disorders: results from the Zurich cohort study. Arch Gen Psychiatry.

[CR42] Molz AR, Black CL, Shapero BG, Bender RE, Alloy LB, Abramson LY (2013). Aggression and impulsivity as predictors of stress generation in bipolar spectrum disorders. J Affect Disord.

[CR43] Nery FG, Hatch JP, Monkul ES, Matsuo K, Zunta-Soares GB, Bowden CL, Soares JC (2013). Trait impulsivity is increased in bipolar disorder patients with comorbid alcohol use disorders. Psychopathology.

[CR44] Nurnberger JI, Blehar MC, Kaufmann CA, York-Cooler C, Simpson SG, Harkavy-Friedman J, Severe JB, Malaspina D, Reich T (1994). Diagnostic interview for genetic studies. Rationale, unique features, and training. NIMH Genetics Initiative. Arch Gen Psychiatry.

[CR45] Peterson CB, Thuras P, Ackard DM, Mitchell JE, Berg K, Sandager N, Wonderlich SA, Pederson MW, Crow SJ (2010). Personality dimensions in bulimia nervosa, binge eating disorder, and obesity. Compr Psychiatry.

[CR46] Petry NM, Barry D, Pietrzak RH, Wagner JA (2008). Overweight and obesity are associated with psychiatric disorders: results from the National Epidemiologic Survey on Alcohol and Related Conditions. Psychosom Med.

[CR47] Potash JB, Chiu YF, MacKinnon DF, Miller EB, Simpson SG, McMahon FJ, McInnis MG, DePaulo JR (2003). Familial aggregation of psychotic symptoms in a replication set of 69 bipolar disorder pedigrees. Am J Med Genet B Neuropsychiatr Genet.

[CR48] Potash JB, Toolan J, Steele J, Miller EB, Pearl J, Zandi PP, Schulze TG, Kassem L, Simpson SG, Lopez V, MacKinnon DF, McMahon FJ, NIMH Genetics Initiative Bipolar Disorder Consortium (2007). The bipolar disorder phenome database: a resource for genetic studies. Am J Psychiatry.

[CR49] Revah-Levy A, Speranza M, Barry C, Hassler C, Gasquet I, Moro M-R, Falissard B (2011). Association between Body Mass Index and depression: the "fat and jolly" hypothesis for adolescents girls. BMC Public Health.

[CR50] Saunders EF, Zhang P, Copeland JN, McInnis MG, Zollner S (2009). Suggestive linkage at 9p22 in bipolar disorder weighted by alcohol abuse. Am J Med Genet B Neuropsychiatr Genet.

[CR51] Saunders EF, Fitzgerald KD, Zhang P, McInnis MG (2012). Clinical features of bipolar disorder comorbid with anxiety disorders differ between men and women. Depress Anxiety.

[CR52] Schag K, Schonleber J, Teufel M, Zipfel S, Giel KE (2013). Food-related impulsivity in obesity and Binge Eating Disorder - a systematic review. Obes Rev.

[CR53] Seixas C, Miranda-Scippa A, Nery-Fernandes F, Andrade-Nascimento M, Quarantini LC, Kapczinski F, Oliveira IR (2012). Prevalence and clinical impact of eating disorders in bipolar patients. Revista brasileira de psiquiatria (Sao Paulo, Brazil: 1999).

[CR54] Simon NM, Otto MW, Wisniewski SR, Fossey M, Sagduyu K, Frank E, Sachs GS, Nierenberg AA, Thase ME, Pollack MH (2004). Anxiety disorder comorbidity in bipolar disorder patients: data from the first 500 participants in the Systematic Treatment Enhancement Program for Bipolar Disorder (STEP-BD). Am J Psychiatry.

[CR55] Wildes JE, Marcus MD, Fagiolini A (2007). Eating disorders and illness burden in patients with bipolar spectrum disorders. Compr Psychiatry.

[CR56] Wildes JE, Marcus MD, Fagiolini A (2008). Prevalence and correlates of eating disorder co-morbidity in patients with bipolar disorder. Psychiatry Res.

[CR57] Young RC, Biggs JT, Ziegler VE, Meyer DA (1978). A rating scale for mania: reliability, validity and sensitivity. Br J Psychiatry.

